# Assessment of Surfactant Protein A (SP-A) dependent agglutination

**DOI:** 10.1186/1471-2466-10-59

**Published:** 2010-11-22

**Authors:** Stefanie M Heinrich, Matthias Griese

**Affiliations:** 1 Dr. von Haunersches Kinderspital, University of Munich, Lindwurmstr. 4a, D-80337 Munich, Germany

## Abstract

**Background:**

Monomers of the collectin surfactant associated protein-A (SP-A) are arranged in trimers and higher oligomers. The state of oligomerization differs between individuals and likely affects SP-A's functional properties. SP-A can form aggregates together with other SP-A molecules. Here we report and assess a test system for the aggregate forming properties of SP-A in serum and broncho-alveolar lavage samples.

**Methods:**

Anti-SP-A antibodies fixed to latex beads bound SP-A at its N-terminal end and allowed the interaction with other SP-A molecules in a given sample by their C-terminal carbohydrate recognition domain (CRD) to agglutinate the beads to aggregates, which were quantified by light microscopy.

**Results:**

SP-A aggregation was dependent on its concentration, the presence of calcium, and was dose-dependently inhibited by mannose. Unaffected by the presence of SP-D no aggregation was observed in absence of SP-A. The more complex the oligomeric structure of SP-A present in a particular sample, the better was its capability to induce aggregation at a given total concentration of SP-A. SP-A in serum agglutinated independently of the pulmonary disease; in contrast SP-A in lung lavage fluid was clearly inferior in patients with chronic bronchitis and particularly with cystic fibrosis compared to controls.

**Conclusions:**

The functional status of SP-A with respect to its aggregating properties in serum and lavage samples can be easily assessed. SP-A in lung lavage fluid in patients with severe neutrophilic bronchitis was inferior.

## Background

Pulmonary surfactant covers the alveoli and terminal airspaces as a complex film made of lipids (90% by mass) and proteins. In the lungs, SP-A is the most abundant surfactant protein by weight. SP-A belongs like SP-D to a family of innate host defence proteins termed collectins because of the presence of a collagenous and a lectin-like domain [1].

The SP-A monomer consists of 228 amino acids and has a molecular weight of 26-32 kDa. Four domains can be differentiated in the primary structure [2]. The N-terminus is followed by the collagen-like region which is linked to the globular head by the neck. SP-A accumulates *in vivo *predominantly as octadecamers composed of six trimeric subunits forming a flower bouquet-like structure [3,4]. The C-terminus with its globular domain contains the recognition site for carbohydrates (CRD). The intermolecular disulfide bonds at the N-terminal end enable the aggregation of trimers to higher oligomers.

In these formations SP-A participates in many physiological functions, including the interaction of the CRD with microorganisms, lectins or other molecules with attached sugar sequences. The CRD is also a ligand for phospholipids [5], involved in the regulation of the surfactant metabolism and phospholipid aggregation [6,7], binds to macrophage membrane proteins [8] and to type-II pneumocytes [9]. On the other hand SP-A can interact with lipids through its collagen like domain. For example, SP-A binds to dipalmitoylphosphatidylcholine (DPPC), the major surfactant phospholipid [10,11]. Furthermore SP-A was found to have direct effects on the survival of Gram-negative bacteria through mechanisms leading to increased permeability of the bacterial cell membrane [12]. SP-A also affects a variety of immune cell functions (reviewed in [13-15]) including alveolar macrophages [16], neutrophils, lymphocytes and dendritic cells [17].

In solution SP-A can self-agglutinate in presence of calcium via its CRD [18]. The threshold concentration of Ca^2+ ^required was 0.5 mM for different species [18,19]. However, the presence of trace amounts of Ca^2+ ^was enough to induce SP-A self-agglutination in physiological-ionic strength buffers at 37°C [20,21]. As shown by Palaniyar et al., 1998 [22] SP-A self-associates further into more ordered configuration of reversible, calcium-dependent supraquaternary structures. The supraquaternary structural form, in turn, forms extensive protein networks when it interacts with phospholipid monolayers [22], possibly preventing collapse of the surfactant lipid films in the presence of protein inhibitors, as they occur in several disease conditions, including adult respiratory distress syndrome or cystic fibrosis [23]. In addition to these biophysical effects, the state of aggregation of SP-A may be important for some of the other effects, especially those on the functions of immune cells [21]. Soluble SP-A had no effect on macrophage production of reactive oxygen species but did stimulate reactive oxygen production when SP-A was multivalently presented after adherence to a surface [24,25]. Therefore the ability of SP-A to form SP-A self-associates, e.g. calcium-dependent supraquaternary SP-A structures, may be a key variable of SP-A present in physiological fluids. Thus a laboratory test is highly desirable which assesses the capability of SP-A present in a given sample to form aggregates. Such an assay was developed by binding of SP-A via its N-terminus to latex beads, so the presenting SP-A CRDs could interact with each other and form visible agglutinates. Further SP-A present in serum and BAL samples from patients was analyzed. Here we describe the characteristics of the test and the functional properties of SP-A in samples from healthy controls, chronic bronchitis patients and patients with cystic fibrosis to induce SP-A self-association.

## Methods

### Subjects

Serum samples from four healthy individuals (mean age 20 years, 3 female) were used for the establishment of the agglutination assay (see below), as described in the legends to the figures in detail. For the investigation of agglutination in patients with different diseases we used samples from 10 patients with cystic fibrosis (2 patients infected with *Pseudomonas aeruginosa*), 10 subjects with chronic bronchitis and 7 additional healthy controls. The clinical details of the subjects are depicted in Table 1. Written informed consent from the subjects was obtained and the experimental research reported was approved by our institutional ethics committee (EK65/96).

**Table 1:** Clinical data

Clinical characteristics	Cystic fibrosis (n = 10)	Chronic bronchitis (n = 10)	Controls (n = 11)
**Age**	14 ± 3 (10)	4 ± 4 (10)	12 ± 8 (11)

**Gender (% female)**	6 (60)	8 (80)	3 (27)

**BMI %, (n)** **(overweight/normal/underweight)**	40/30/30 (4/3/3)	0/100/0 (0/10/0)	0/100/0 (0/11/0)

**Fev_1 _(% pred.)**	97 ± 31 (10)	109 ± 15 (3)	118 (1)

**SP-A level in serum (ng/ml)**	26 ± 10 (10)	28 ± 24 (2)	17 ± 4 (10)

**SP-A level in BAL (ng/ml)**	4993 ± 2984 (10)	4094 ± 1363 (3)	6645 ± 5354 (11)

**Neutrophils in BAL (%)**	42 ± 29 (10)	9 ± 9 (5)	2.5 ± 4 (4)

Data are means ± SEM and range of (n) subjects.

### BAL

BAL was performed as described [26] with 4 × 1 ml/kg body weight normal saline warmed to body temperature. The first aliquot of the recovered BAL fluid was treated separately; all other samples were pooled for analysis. The total cell count was measured in a hemocytometer; the differential cell count was assessed from cytoprep slides. Aliquots of the cell free BAL supernatant of the pooled BAL sample were used for the analysis of total protein.

### Gel chromatography

To determine structural differences in BAL and serum between the study populations a gel chromatography method with a superose 6 column was chosen as described [27]. In this method SP-A was separated with regard to its oligomerization form. 1 ml BAL or 500 μl serum were loaded. The larger the oligomers the earlier they were eluted. After gel chromatography the SP-A amount of the eluted fractions (800 μl) was determined by Slot-Blot. The void volume of the column was about 6.8 ml eluting between fractions 8 and 9.

### Slot blot

A slot blot was used to quantify SP-A concentration. 200 μl aliquots of the chromatography fractions and 100 μl standard recombinant human SP-A (gift of Dr. Wolfram Steinhilber, Nycomed, Konstanz, Germany) were applied onto blot membranes in a Slot-Blot gadget (BioRad, Munich, Germany). The membrane was blocked with blocking buffer (2 g TWEEN, 6 g Fish gelatine, 200 ml TBS: *Tris*-buffered saline, 50 mM *Tris*; 150 mM NaCl) for 3 h. After washing 3 times, the membrane was incubated with a polyclonal recombinant rabbit-anti-human SP-A antibody (Nycomed, Konstanz, Germany) and 1% bovine serum albumin (BSA, Paesel+Lorei, Duisburg, Germany) overnight. Following 3 washes for 10 min with TBS-T (TBS with 0.05% Tween20) and incubation with the second goat-anti-rabbit antibody (1:10,000, Biozol, Eching, Germany) and 1% BSA for 2 h, the membrane was washed finally. For signal detection the membrane was incubated for 1 min in a mixture of 500 μl of each reagent from Super Signal West Dura (Pierce, Bonn, Germany) and 1 ml of each reagent from ECL Western Blot Detection Reagents (GE Healthcare, Munich, Germany). Signals were measured with a DIANA III camera (raytest, Straubenhardt, Germany) by 15 min duration of exposure and evaluated by AIDA Software (raytest, Straubenhardt, Germany). The reproducibility of the slot-blot assay was good, as determined by the coefficient of variation at the highest concentration (41.6 ng/ml; rel CV 6.1%) of the standard curve.

### SP-A self-agglutination measurement

**Figure 1:**
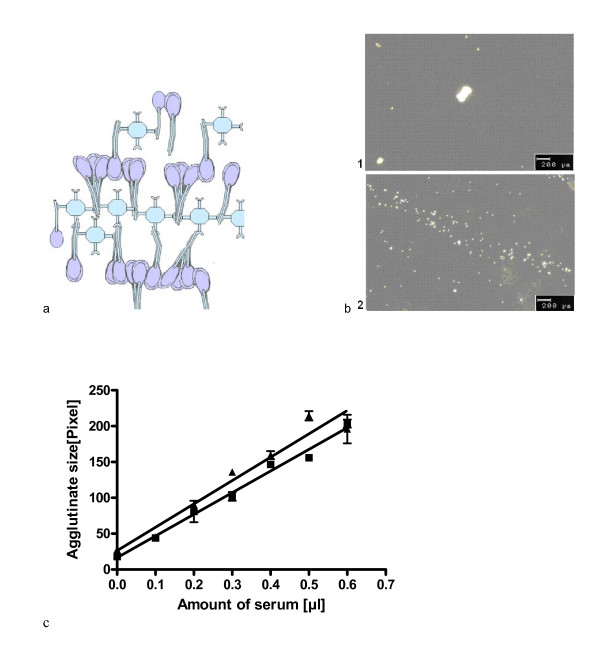
**SP-A self-agglutination assay**. a) The figure shows a scheme of the SP-A self-agglutination-assay. The streptavidin beads were coupled to biotinylated rabbit anti-goat antibodies which bound goat anti-human SP-A antibodies. These anti-SP-A antibodies bound SP-A at its N-terminal end, so SP-A could self-agglutinate by its CRD. The scheme attempts to illustrate the components of the reactants, but does not render how the molecules bind exactly. b) The microscope pictures with a magnification of 10 times were taken under a light microscope. Picture a shows an agglutinate while picture b illustrates beads without agglutination. c) The graph illustrates the SP-A dependency of the bead agglutination. The agglutinate size is plotted against the amount of serum containing SP-A which was incubated with the beads, anti-goat antibody and goat-anti-human antibody in a buffer with calcium ions. Two different sera were used (Black triangle, SP-A concentration 20 ng/ml, sample 2 and balck square, SP-A concentration 21 ng/ml, sample 1) in the experiments which were repeated three times. The final SP-A amount was in 0.6 μl serum about 16 pg.

An assay was designed to assess the ability of SP-A in serum and broncho-alveolar lavage samples to agglutinate. Schematically, anti-SP-A antibodies which bind SP-A at its N-terminal end were coupled to 0.05 μm beads, so that the bound SP-A was able to interact by its C-terminal CRD with other SP-A molecules in a given sample. This causes agglutination of the beads forming larger aggregates, which were observed by light microscopy (Figure 1).

To achieve this, 50 μl (1.82 × 10^10 ^beads/ml) of a carboxylate-modified, streptavidin-labeled latex bead mixture (Sigma Aldrich, Munich, Germany) was incubated with 200 μl biotinylated rabbit anti-goat antibody (1.3 mg/ml, Dianova, Hamburg, Germany) for 18 h at 23°C in 0.2 ml Eppendorf tubes (Eppendorf, Hamburg, Germany). The mixture was washed 2 times with Hank solution (8 g/l NaCl, 1 g/l glucose, 0.4 g/l KCl, 0.35 g/l NaHCO_3_, 0.14 g/l CaCl_2_, 0.1 g/l MgCl_2_·6H_2_O, 0.06 g/l Na_2_HPO_4_·2H_2_O, 0.06 g/l KH_2_PO_4 _and 0.06 g/l MgSO_4_·7H_2_O, pH 7.4; Apotheke Innenstadt, University Munich, Germany) and resuspended in 600 μl Hank solution. Then 5 μl of the samples (final concentration of SP-A derived from the peak fractions was 100 ng/ml SP-A) were incubated with 2 μl bead suspension at 6.5 mM Ca^2+^, 3 μl pure water and 0.01 μg of the anti-SP-A antibody (N19, Santa Cruz, Heidelberg, Germany) for 3 h at 4°C on a microscope slide (Menzel, Braunschweig, Germany) under a cover slip (Menzel, Braunschweig, Germany). To avoid non-specific agglutination of the SP-A, all assays were done in the presence of 0.1% (v/v) Triton X-100. Under the light-optical microscope (Axioskop, Zeiss, Aalen, Germany) the biggest, pure bead-agglutinate was searched and photographed. The picture was viewed by the software Adobe Photoshop (Adobe, Munich, Germany) at a size of 764×573 pixels. Then, the size of the determined agglutinate was measured in pixel by drawing a square around it (Figure 1b).

For evaluating the reproducibility and sensitivity of the assay 2 serum samples from healthy individuals (SP-A concentration in serum was 20 ng/ml for both) were tested by using serum volumes from 0 to 0.6 μl in 0.1 μl steps (Figure 1c). The tests were repeated 3 times and the coefficient of variation was between 1-5%.

## Results

### Characteristics of the assay to assess SP-A self-agglutination

First the specificity for SP-A, and not for SP-D, and the dependency of the assay on the presence of a complete binding chain consisting of the beads coupled with the anti-goat antibody, and the anti-human-SP-A antibody was shown. Only the addition of SP-A (10 μl of a 100 ng/ml recombinant human SP-A) and the presence of all components induced agglutination (Table 2). Recombinant human SP-D had no effect on agglutination (Table 2).

**Table 2:** Components necessary to induce SP-A self-agglutination under the assay conditions used

Condition	Reagent				Agglutination
	**SP-A**	**SP-D**	**Goat-anti-human-SP-A antibody**	**Anti-goat antibody**	

**1**	**+**	**-**	**+**	**+**	**+**

**2**	**-**	**+**	**+**	**+**	**-**

**3**	**+**	**-**	**-**	**+**	**-**

**4**	**+**	**-**	**+**	**-**	**-**

**5**	**-**	**-**	**+**	**+**	**-**

The table shows five different conditions of the bead-assay. The beads were incubated alternatively with anti-goat antibody, goat-anti-human antibody, SP-A (1 ng recombinant human standard, final concentration 100 ng/ml) or SP-D (1 ng recombinant human standard, final concentration 100 ng/ml) in a buffer containing calcium ions. The "+" means that the reagent was used. After incubation the mixture was analyzed by light microscopy and agglutination was only detected under condition 1 when SP-A was added. The experiments were repeated three times.

**Figure 2:**
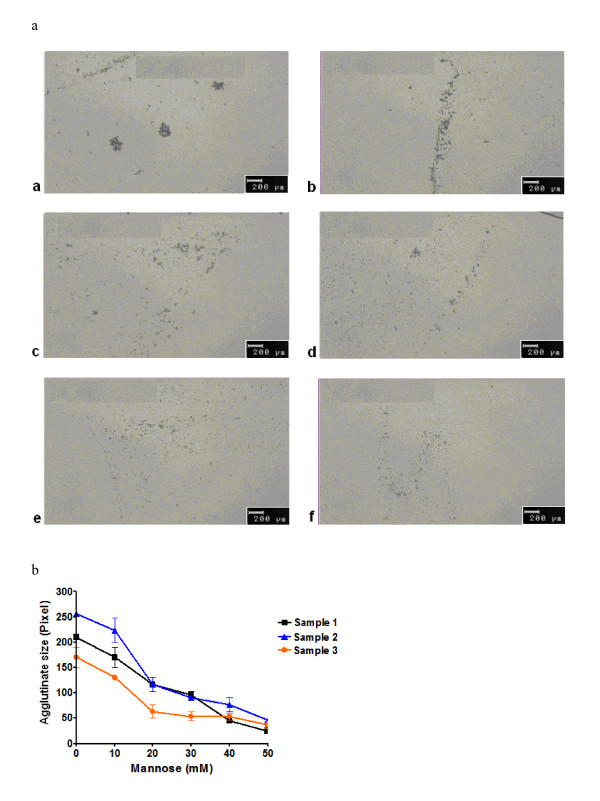
**Carbohydrate-dependency of the agglutination of beads by SP-A**. a) The pictures were taken under a light microscope by a 10 time magnification. Anti-goat antibody coated beads were incubated with goat anti-human SP-A antibody, 5 μl serum and an increasing amount of mannose (a: 0 mM mannose; b: 10 mM mannose; c: 20 mM mannose; d: 30 mM mannose; e: 40 mM mannose; f: 50 mM mannose). The agglutinate size decreased corresponding to an increasing mannose concentration. b) The figure shows a graph of the agglutinate size out of serum samples from three different test persons under varying mannose concentrations in a buffer containing calcium ions. SP-A concentration was 21 ng/ml in sample 1, 20 ng/ml in sample 2, and 20 ng/ml in sample 3. The higher the mannose concentration was, the smaller were the bead agglutinates. The experiments were repeated five times.

**Figure 3:**
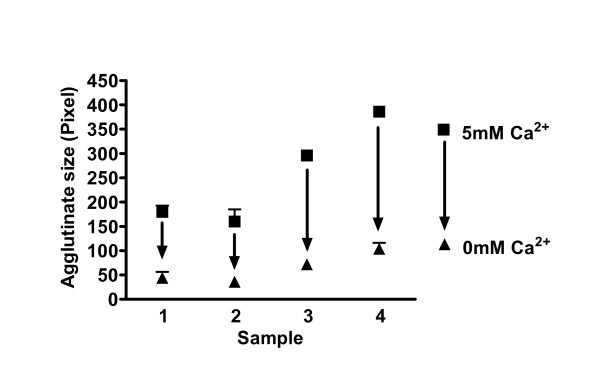
**Calcium-dependency of the bead-assay**. The graph displays the dependency on calcium ions of the bead agglutination caused by SP-A. Serum samples of four test subjects (SP-A concentrations see legends to figs. 1 and 2) were incubated with beads, anti-goat antibody, goat-anti-human antibody in a buffer containing calcium ions first without EDTA (black square) and then with 50 mM EDTA (black triangle). Each experiment was repeated five times. Removal of calcium by chelation with EDTA leads to smaller bead agglutinates.

Next the calcium and carbohydrate dependency of the assay was assessed (Table 3). Results demonstrated that an increasing concentration of mannose as well as the absence of calcium inhibited the formation of large agglutinates (Figure 2 and 3). On the other hand serum and its many factors including SP-D, tested with serum from patients with and without SP-D, had no effect on SP-A self-agglutination (Tables 2 and 3). These data confirmed the specificity of our assay for SP-A and not SP-D and its utility for BAL and serum samples.

**Table 3:** Tests for SP-A self-agglutination-assay dependency

Beads	Goat-anti-human-SP-A antibody	Anti-goat antibody	Serum	Ca^2+^	Agglutination
+	+	+	+	-	-

+	+	+	+	+	+

+	+	+	-	+	-

+	+	+	-	-	-

+	-	+	+	+	-

+	-	-	+	+	-

-	+	+	+	+	-

+	+	+	SP-D	-	-

+	+	+	SP-D	+	-

+	+	+	-	+ with Mannose	-

+	+	+	+	+ with Mannose	-

+	+	+	+/without SP-D	+	+

+	+	+	+/+Triton X	+	-

The table shows twelve conditions of the bead assay with varying reagents: beads, anti-goat antibody, goat-anti-human antibody, human serum and calcium ions (Ca^2+^). The "+" means that the reagent was used for incubation. Without SP-A and/or calcium ions in serum, and also with Mannose no agglutination was detected. The experiments were repeated three times. For the last condition a concentration of 0.1% (v/v) Triton X-100 was used (0.3 μl Triton X-100 in 1 ml pure water, 3 μl of this water were used for the assay). A final concentration of 100 ng/ml SP-A (1 ng) in test serum and 100 ng/ml SP-D standard (1 ng recombinant human SP-D) were used.

### Analysis of serum samples

In 10 patients with cystic fibrosis the serum SP-A agglutination ability was measured. The size of the agglutinates was 1364 pixel ± 616 (range 667 to 2862) and did not significantly differ from that obtained with the corresponding lavage fluid (1059 ± 346, range 560 to 1610).

Because the size of the SP-A agglutinates varied between individuals and it is known that SP-A is present in different oligomeric forms, we assumed that differences in the structural complexity may be responsible for the variation observed. Therefore, we further analyzed the SP-A self-agglutination ability of the fractions derived from gel chromatography, containing different oligomeric structural forms of SP-A. Gel chromatography usually yields three peaks containing the main different oligomeric structures: an initial peak represented by fraction 10 (F10) containing the largest forms, i.e. octadecamers (18 mers) or more complex structural forms of SP-A, followed by the second peak represented by fraction 15 (F15), containing 6 to 12 mers, and finally the third peak represented by fraction 20 (F20) containing the smallest forms, i.e. di- and trimers or monomers. Using the SP-A self-agglutination assay, each fraction was analyzed adjusted with respect to the SP-A concentration.

**Figure 4:**
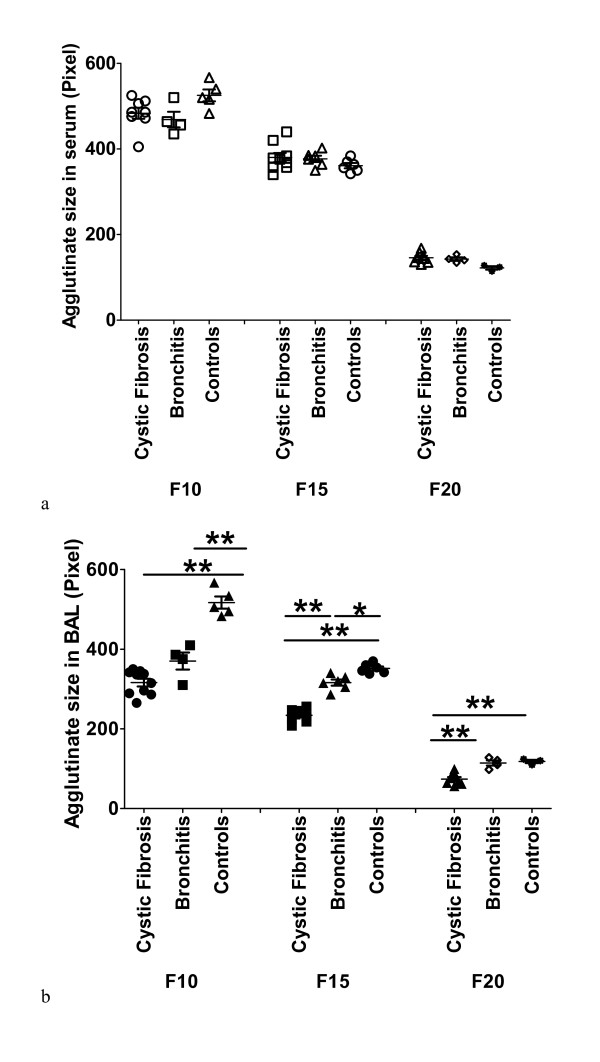
**SP-A self-agglutination and fractions**. The graphs show the self-agglutination ability (y-axis) of different SP-A structures derived from BAL (b) and serum (a) (x-axis) of the study populations. The streptavidin beads were coupled to biotinylated rabbit anti-goat antibodies which bound goat anti-human SP-A antibodies. These anti-SP-A antibodies bound SP-A at its N-terminal end, so SP-A could self-agglutinate by its CRD. The SP-A amount was adjusted to 1 ng (final concentration 100 ng/ml). All experiments were analyzed by One way ANOVA. * stands for p < 0.05, ** for p < 0.01 and *** for p < 0.001. In BAL and serum of all three study populations (10 CF, 10 Bro, 7 C) there was a significant difference between the self-agglutination ability of the different structures (F10, F15 and F20). The octadecamers or more complex structures showed the best self-agglutination ability followed by the hexameric structures and then the dimers/trimers. Comparing the self-agglutination ability between the study groups, in all BAL fractions the SP-A oligomers derived from controls agglutinated significantly better than the SP-A oligomers derived from patients, while SP-A from bronchitis patients agglutinated still better than from CF patients; except for F20 of bronchitis samples, which showed the same self-agglutination ability as controls. In contrast to BAL in serum there was no difference between the study populations regarding the self-agglutination ability of the different fractions (p > 0.05). Comparing the agglutination ability between BAL and serum, only in the patient populations (CF and Bro) all SP-A structures derived from serum fractions agglutinated significantly better than the corresponding structures derived from BAL. While in CF patients this difference was highly significant for all structures in the bronchitis study population the significance decreased from fraction 10 over fraction 15 to fraction 20.

In sera of all three study populations, CF, bronchitis and controls, there was a significant difference regarding the SP-A self-agglutination ability between the fractions (Figure 4a). There was a steady decrease in agglutinate size from SP-A octadecamers or more complex structures, over the hexamers to the fraction of dimers and trimers. Of interest, in serum the SP-A agglutinate sizes of the fractions were equal comparing the sample groups (Figure 4a).

### Analysis of BAL samples

In contrast to serum, the SP-A derived from patient BAL samples indicated a major impact on the ability of the fractions to induce SP-A dependent agglutination. For all BAL fractions the SP-A oligomers derived from controls agglutinated significantly better than the SP-A oligomers derived from patients. Also samples from the bronchitis population agglutinated better than those from CF patients (Figure 4b).

Comparing the SP-A self-agglutination ability of the fractions between BAL and serum within the groups of CF and bronchitis patients there was also a significant difference (Figure 4a and 4b), but not in controls. Regarding the two disease groups SP-A structures in serum agglutinated significantly better than the corresponding structures in BAL.

To test if qualitative changes within the SP-A from a specific patient might be responsible for the observed effects, certain gel-chromatography fractions of SP-A present in different patients at different levels, were isolated, the concentration adjusted and tested for their capacity to agglutinate; no differences were found (data not shown). This result suggests that the relative composition of the whole sample with respect to the different oligomeric forms is responsible for its agglutination activity and not potential differences among the oligomeric forms within a particular fraction.

In conclusion, these results showed that SP-A in serum had a better functional ability than SP-A in BAL from patients with CF or chronic bronchitis, but not from controls. Also, in BAL of patients with a lung disease the self-agglutination ability of SP-A was diminished compared to controls. In all three study groups, the more complex SP-A oligomers were, the better was their self-agglutination ability.

## Discussion

Systematic evaluation of the conditions to induce microscopically visible aggregation of SP-A showed the dependency of the test on the concentration of SP-A added into the reaction, the presence of calcium, and dose-dependent inhibition by mannose. It was established that SP-D alone, serum without SP-A or without SP-A and SP-D did not induce aggregation. Further we showed that the more complex the oligomeric structure of SP-A present in a particular sample was, the better was its capability to induce aggregation at a given total concentration of SP-A. Comparing the SP-A agglutination ability of the fractions no differences were observed for SP-A derived from serum independent of the pulmonary disease state. In contrast, in BAL fluid, it was clearly shown that patients with chronic bronchitis had some, and those with cystic fibrosis had the most severe impairments of SP-A capability to induce aggregation. Thus, the agglutination assay was measuring the ability of SP-A molecules to aggregate as an active process induced in the assay. This ability was mainly a reflection of the oligomeric structure of SP-A in the samples, further of the origin of the samples and the disease state.

The strength of this approach is the specific measurement of the CRD binding of SP-A by a very simple and fast method and the possibility for *ex vivo *analysis in very small volume samples of serum and BAL. There is also no step necessary to isolate the SP-A before the measurement. As the goal of the study was to establish the assay, the patient group sizes were relatively small. However, the results were highly reproducible. Further studies in larger populations are desirable and are underway.

Our data confirm the calcium-dependency of SP-A self-agglutination by its CRD, previously reported [18,21,28]. Additionally, they are in agreement with previous authors supporting the thesis that the SP-A self-agglutination capability may be important for its function [24,25]. However, to what extent SP-A self-agglutination capability correlates with anti-inflammatory effects [29] or the many other modulatory effects in different cell types, needs to be determined in studies investigating several of these additional variables in parallel.

We found that also smaller SP-A structures can agglutinate, but as expected from *in vitro *data, to a lesser extent than octadecamers. This data is important as for the first time *ex vivo *SP-A from clinically well defined patients with other lung diseases than pulmonary alveolar proteinosis (PAP) was used extensively. SP-A from PAP patients differs from normal SP-A, as the large molecular structures are mostly non-reducible [30,31]. Furthermore, functional discrepancies were found to non-proteinosis SP-A [32], as well as between recombinant and native SP-A forms with respect to their self-association ability [33].

SP-D is another lung collectin, also present in the alveolar space and in serum, and shows structural similarities to SP-A. SP-D monomers form trimers and four trimers assemble to a cruciform structure with CRDs at their end [34]. Similar to SP-A, SP-D can agglutinate micro-organisms and bind to cells [35]. In previous studies we have also assessed its functional ability in samples from patients with various pulmonary diseases [36]. Of importance we demonstrated here no influence of SP-D on our system.

The data clearly show a local pulmonary deficiency of SP-A dependent agglutination in the pulmonary diseases investigated, whereas no abnormalities were seen in systemic circulation.

In future studies it will be important to investigate the correlation of this assay with other functional properties of SP-A, and more importantly the correlation with lung function and long term disease outcome variables, in order to determine its role as a surrogate marker and potential modulator of the clinical course.

## Conclusions

In summary, we describe the characteristics of the test system to assess functional properties of SP-A in serum and lavage samples. Samples from healthy controls, bronchitis patients and patients with cystic fibrosis induced SP-A self-association; the more complex the SP-A structures were, the better were the self-agglutination abilities. Comparing the functional capabilities of corresponding SP-A structures separated from serum and BAL, in the two lung disease groups, BAL samples had inferior SP-A self-agglutination abilities. This clearly showed a strong structure - function relation of SP-A for normal subjects and some disease states with altered SP-A.

## Abbreviations

SP-A; SP-D; CRD

## Competing interests

The authors declare that they have no competing interests.

## Authors' contributions

SMH carried out the microscopic and biochemical studies. MG conceived the study. MG and SMH designed the experiments, performed the statistical analysis and drafted the manuscript. All authors read and approved the final manuscript.

## Pre-publication history

The pre-publication history for this paper can be accessed here:

http://www.biomedcentral.com/1471-2466/10/59/prepub
